# Quantum internet using code division multiple access

**DOI:** 10.1038/srep02211

**Published:** 2013-07-17

**Authors:** Jing Zhang, Yu-xi Liu, Şahin Kaya Özdemir, Re-Bing Wu, Feifei Gao, Xiang-Bin Wang, Lan Yang, Franco Nori

**Affiliations:** 1 CEMS, RIKEN, Saitama, 351-0198, Japan; 2 Department of Automation, Tsinghua University, Beijing 100084, P. R. China; 3 Center for Quantum Information Science and Technology, TNList, Beijing 100084, P. R. China; 4 Institute of Microelectronics, Tsinghua University, Beijing 100084, P. R. China; 5 Electrical and Systems Engineering, Washington University, St. Louis, Missouri 63130, USA; 6 Department of Physics, Tsinghua University, Beijing 100084, P. R. China; 7 Physics Department, The University of Michigan, Ann Arbor, MI 48109-1040, USA

## Abstract

A crucial open problem inS large-scale quantum networks is how to efficiently transmit quantum data among many pairs of users via a common data-transmission medium. We propose a solution by developing a quantum code division multiple access (q-CDMA) approach in which quantum information is chaotically encoded to spread its spectral content, and then decoded via chaos synchronization to separate different sender-receiver pairs. In comparison to other existing approaches, such as frequency division multiple access (FDMA), the proposed q-CDMA can greatly increase the information rates per channel used, especially for very noisy quantum channels.

QUantum networks for long distance communication and distributed computing require nodes which are capable of storing and processing quantum information and connected to each other via photonic channels^1^,^2^,^3^. Recent achievements in quantum information^4^,^5^,^6^,^7^,^8^,^9^,^10^ have brought quantum networking much closer to realization. Quantum networks exhibit advantages when transmitting classical and quantum information with proper encoding into and decoding from quantum states^11^,^12^,^13^,^14^,^15^,^16^,^17^. However, the efficient transfer of quantum information among many nodes has remained as a problem yet to be solved^18^,^19^,^20^,^21^,^22^,^23^,^24^, which becomes more crucial for the limited-resource scenarios in large-scale networks. Multiple access, which allows simultaneous transmission of multiple quantum data streams in a shared channel, may provide a remedy to this problem.

Popular multiple-access methods in classical communication networks include time-division multiple-access (TDMA), frequency-division multiple-access (FDMA), and code-division multiple-access (CDMA). See Fig. 1 for an illustration of different multiple-access methods. In TDMA, different users share the same frequency but transmit on different time slots, but timing synchronization and delays become serious problems in large-scale networks. In FDMA, different users share the same time slots but operate on different frequency bands. However, only a narrow band of the data transmission line has a low leakage rate and the bands assigned to different users should be sufficiently separated to suppress interference. Unlike TDMA and FDMA, CDMA utilizes the entire spectrum and time slots to encode the information for all users, while distinguishes different users with their own unique codes. Therefore, CDMA is adopted as the key technology of the currently-used third generation mobile communication systems, and can accommodate more bits per channel use^25^ compared with TDMA and FDMA. It has achieved great success in commercial applications of classical communications.

Although FDMA has already been used in quantum key distribution networks^26^,^27^,^28^,^29^,^30^, to the best of our knowledge, CDMA has not yet been applied in quantum networks and internet^1^. A q-CDMA network would require that the states sent by each transmitting node of the quantum network are encoded into their coherent superposition before being sent to the common channel, and the quantum information for each of the intended receiving node is coherently and faithfully extracted by proper decoding at the end of the common channel. This, however, is not a trivial task but rather a difficult one.

In this paper, we propose a q-CDMA method via chaotic encoding and chaos synchronization among senders and receivers, which require a quantum channel to transmit quantum superposition states and N classical channels for chaos synchronization to decode the quantum signals at the receiver nodes. It can be seen that the proposed q-CDMA provides higher transmission rates for both classical and quantum information, especially in very noisy channels.

## Results

**Figure 2:**
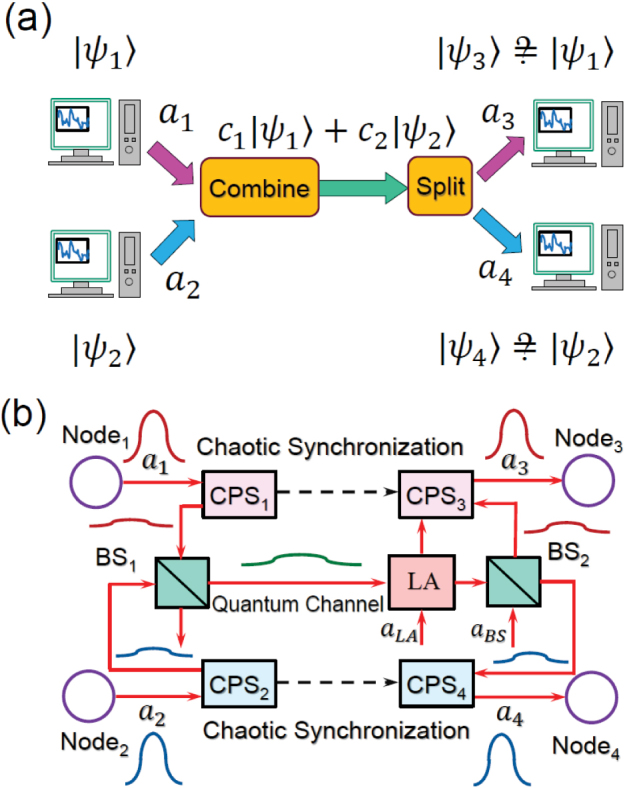
Diagrams of the quantum multiple access networks. (a) Quantum information transmission between two pairs of nodes via a single quantum channel. Quantum states from two senders are combined to form a superposition state and input to the channel. At the receiver side, they are coherently split into two and sent to the targeted receivers. (b) Schematic diagram of the q-CDMA network by chaotic synchronization. Wave packets from the sender nodes are first spectrally broadened by using the chaotic phase shifters CPS_1_ and CPS_2_, and then mixed at a beamsplitter (BS_1_) and input to the channel. After linear amplification (LA) and splitting at the second beamsplitter (BS_2_), individual signals are recovered at the receiver end with the help of CPS_3_ and CPS_4_, which are synchronized with CPS_1_ and CPS_2_, respectively.

To present the underlying principle of our method, we consider the simplest case, where two pairs of sender and receiver nodes communicate quantum information, encoded into quantized electromagnetic fields with the *same* frequencies, via a single quantum channel [see Fig. 2(a)].

The schematic diagram of our strategy is shown in Fig. 2(b). The quantum information sent by the nodes 1 and 2 is first encoded by two chaotic phase shifters CPS_1_ and CPS_2_, whose operation can be modelled by the effective Hamiltonian 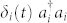, with *δ_i_*(*t*) being time dependent classical chaotic signals and *i* = 1, 2. This encoding spreads the spectral content of the quantum information across the entire spectrum. The two beams are then combined at the 50:50 beamsplitter BS_1_ and transmitted via a common channel to the receivers. At the end of the channel, the quantum signal is first amplified by a phase-insensitive linear amplifier (LA), then divided into two branches by a second 50:50 beamsplitter BS_2_, and finally sent to nodes 3 and 4 through two chaotic phase shifters CPS_3_ and CPS_4_, which are introduced to decode the information by applying the effective Hamiltonian 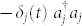, with *j* = 3, 4. Amplifier gain is set as *G* = 4 to compensate the losses induced by the beamsplitters.

The actions of the chaotic devices CPS*_i_*_ = 1,2,3,4_ induce the phase shifts exp [−*iθ_i_*(*t*)], where 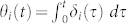. Thus, to achieve faithful transmission between the senders and the receivers, the effects of *δ*_1_(*t*) and *δ*_2_(*t*) on the quantum signals should be minimized in the fields received by the nodes 3 and 4. Intuitively, this could be done by simply adjusting the system parameters such that *δ*_1_(*t*) = *δ*_3_(*t*) and *δ*_2_(*t*) = *δ*_4_(*t*). However, such an approach is impractical, because any small deviation in the system parameters can be greatly amplified by the chaotic motion, making it impossible to keep two chaotic circuits with the same exact parameters and initial conditions. Instead, auxiliary classical channels between senders and the intended receivers can be used to synchronize the chaotic circuit as shown in Fig. 2(b). This classical chaotic synchronization helps to reduce the parameter differences between the chaotic phase shifters and to extract the quantum information faithfully.

### Modelling of quantum CDMA network

Hereafter, for the sake of simplicity, we assume that CPS_1_ (CPS_2_) and CPS_3_ (CPS_4_) have been synchronized before the start of the transmission of quantum information, i.e., *θ*_1_(*t*) = *θ*_3_(*t*) [*θ*_2_(*t*) = *θ*_4_(*t*)]. The whole information transmission process in this quantum network can be described by the input-output relationship 
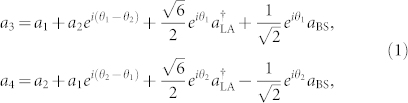
where 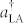 and *a*_BS_ are the creation and annihilation operators of the auxiliary vacuum fields entering the linear amplifier LA and the second beamsplitter BS_2_. For the pseudo-noise chaotic phase-shift *θ_i_*(*t*), we should take an average over this broadband random signal, which leads to 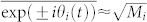 With 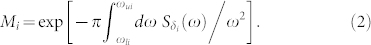
In Eq. (2), 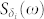 is the power spectrum density of the signal *δ_i_*(*t*), and *ω_li_* and *w_ui_* are the lower and upper bounds of the frequency band of *δ_i_*(*t*), respectively. Equation (1) can then be reduced to 
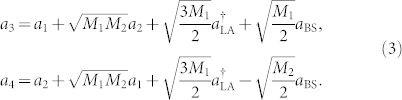
For a chaotic signal with broadband frequency spectrum, the factor *M_i_* is extremely small, and can be neglected in Eq. (3). This leads to *a*_3_ ≈ *a*_1_ and *a*_4_ ≈ *a*_2_, implying efficient and faithful transmission of quantum information from nodes 1 and 2 to nodes 3 and 4, respectively.

In our q-CDMA network, the information-bearing fields *a*_1_ and *a*_2_, having the same frequency *ω_c_*, are modulated by two different pseudo-noise signals, which not only broaden them in the frequency domain but also change the shape of their wavepackets [see Fig. 2(b)]. Thus, the energies of the fields *a*_1_ and *a*_2_ are distributed over a very broad frequency span, in which the contribution of *ω_c_* is extremely small and impossible to extract without coherent sharpening of the *ω_c_* components. This, on the other hand, is possible only via *chaos* synchronization which effectively eliminates the pseudo-noises in the fields and enables the recovery of *a*_1_ (*a*_2_) at the output *a*_3_ (*a*_4_) with almost no disturbance from *a*_2_ (*a*_1_). This is similar to the classical CDMA. Thus, we name our protocol as q-CDMA.

### Quantum state transmission

**Figure 3:**
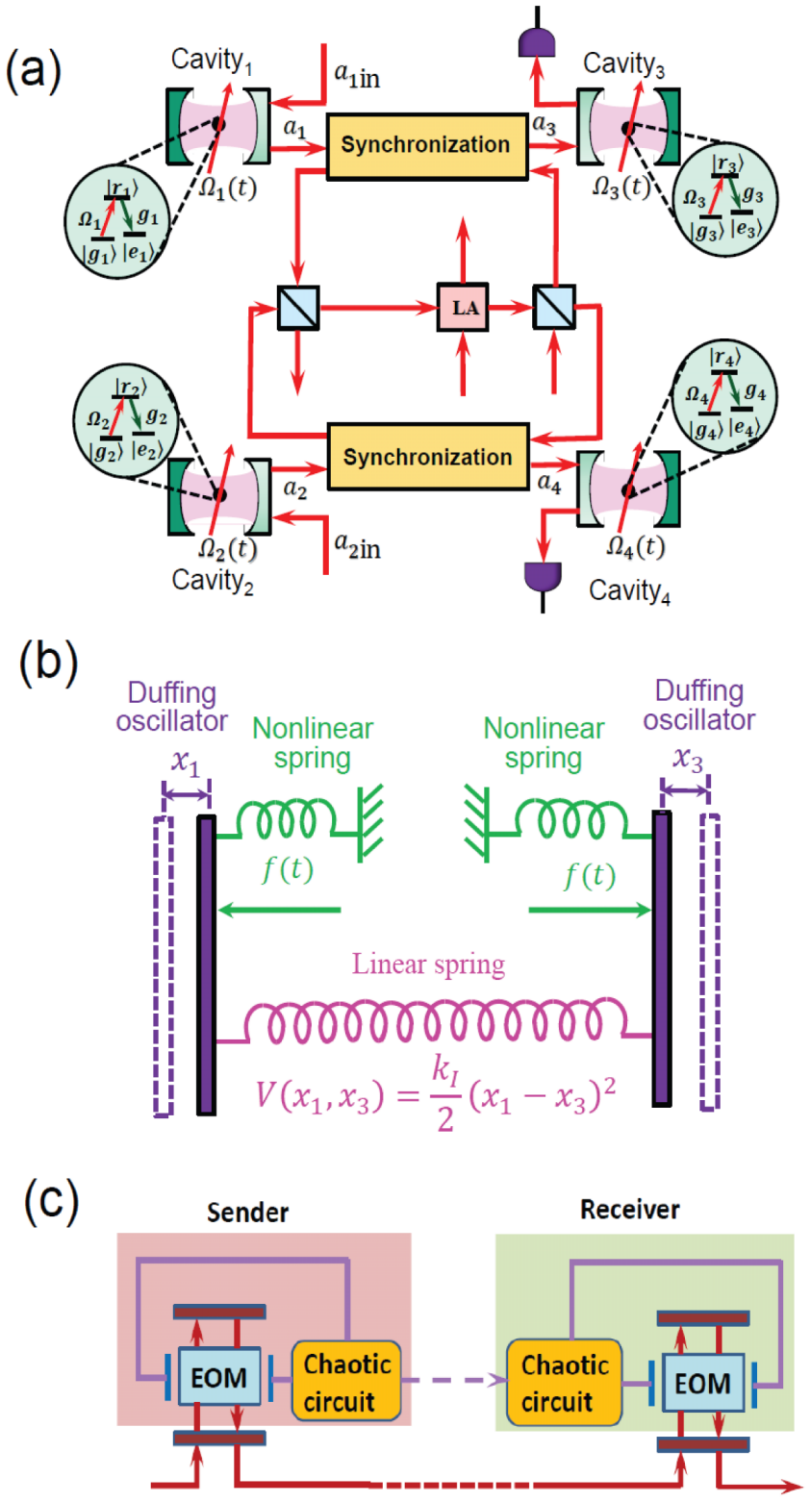
Quantum state transmission over q-CDMA network. (a) The broom-shaped or shovel-shaped purple symbols denote photon detectors. The red arrow inside each (green) cavity denotes the classical driving field with amplitude Ω*_i_*(*t*) (*i* = 1, 2, 3, 4). The green circles denote Λ-type three-level atoms. (b) Schematic diagram of the chaotic synchronization realized by the moving mirrors. (c) Chaotic encoding and decoding by electro-optic modulators.

Let us further study the transmission of qubit states over the proposed q-CDMA network using a concrete model. The qubit states {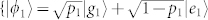, and 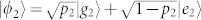, with *p*_1_, *p*_2_ ∈ [0, 1]}, to be transmitted are encoded in the dark states of two Λ-type three-level atoms; i.e., atom 1 in cavity 1 and atom 2, in cavity 2, as shown in Fig. 3(a). The qubit states are transferred to the cavities by Raman transitions and are transmitted over the q-CDMA network. At the receiver nodes, the quantum states are transferred and stored in two Λ-type atoms; i.e., atom 3 in cavity 3, and atom 4 in cavity 4. We assume that the four coupled atom-cavity systems have the same parameters. Let |*g_i_*〉, |*e_i_*〉, and |*r_i_*〉 be the three energy levels of atom *i*. As shown in Fig. 3(a), the |*g_i_*〉 → |*r_i_*〉 and |*e_i_*〉 → |*r_i_*〉 transitions are coupled with a classical control field and a quantized cavity field with coupling strengths Ω*_i_* (*t*) and *g_i_* (*t*). By adiabatically eliminating the highest energy level |*r_i_*〉, the Hamiltonian of the atom-cavity system can be expressed as 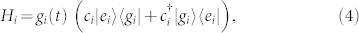
where *c_i_* is the annihilation operator of the *i*-th cavity mode; *g_i_* (*t*) = *g*Ω*_i_* (*t*)/Δ is the coupling strength which can be tuned by the classical control field Ω*_i_* (*t*); and Δ is the atom-cavity detuning. The cavity fields *c_i_* are related to the travelling fields *a_i_* by 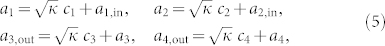
where *κ* is the decay rate of the cavity field; and *a*_1,in_, *a*_2,in_ (both in vacuum states) and *a*_3,out_, *a*_4,out_ are the input and output fields of the whole system, respectively.

The chaotic phase shifters CPS*_i_*_ = 1,2,3,4_ are realized by coupling the optical fields to four driven Duffing oscillators, with damping rates *γ*, described by the Hamiltonian 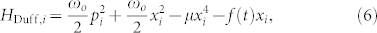
where *x_i_* and *p_i_* are the normalized position and momentum of the nonlinear Duffing oscillators, *ω*_0_/2*π* is the frequency of the fundamental mode, *μ* is a nonlinear constant, and *f*(*t*) = *f_d_* cos (*ω_d_t*) is the driving force. The interaction between the field *a_i_* and the *i*-th Duffing oscillator is given by the Hamiltonian 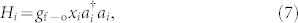
where *g*_f–o_ is the coupling strength between the field and the oscillator. Under the semiclassical approximation for the degrees of freedom of the oscillator, the interaction Hamiltonian *H_i_* leads to a phase factor 
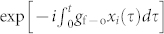
 for the field *a_i_*. To simplify the discussion, we assume that all of the four Duffing oscillators have the same *ω*_0_, *μ*, *f_d_*, and *ω_d_*, but different initial states. Finally, the chaotic synchronization between CPS_1_ (CPS_2_) and CPS_3_ (CPS_4_) is achieved by coupling two Duffing oscillators by a harmonic potential *V* (*x*_1_, *x*_3_) = *k_I_* (*x*_1_ − *x*_3_)^2^/2.

The nonlinear coupling between the optical fields and the Duffing oscillators and the chaos synchronization to achieve the chaotic encoding and decoding could be realized using different physical platforms. For example, in optomechanical systems, the interaction Hamiltonian (7) can be realized by coupling the optical field via the radiation pressure to a moving mirror connected to a nonlinear spring (see Fig. 3(b)). Chaotic mechanical resonators can provide a frequency-spreading of several hundreds of MHz for a quantum signal, and this is broad enough, compared to the final recovered quantum signal, to realize the q-CDMA and noise suppression. Chaos synchronization between different nonlinear mechanical oscillators can be realized by coupling the two oscillators via a linear spring. This kind of synchronization of mechanical oscillators have been realized in experiments^32^, but it is not suitable or practical for long-distance quantum communication. Chaos synchronization with a mediating optical field, similar to that used to synchronize chaotic semiconductor lasers for high speed secure communication^33^, would be the method of choice for long-distance quantum communication. The main difficulty in this method, however, will be the coupling between the classical chaotic light and the information-bearing quantum light. This, on the other hand, can be achieved via Kerr interactions. There is a recent report^34^ that proposes to use Kerr nonlinearity in whispering gallery mode resonators to solve this problem. Another approach for chaotic encoding and chaos synchronization between distant nodes of the network could be the use of electro-optic modulators (EOMs). See, e.g., Fig. 3(c). In this case, the input information-bearing quantum signal is modulated by the EOM driven by a chaotic electrical signal^35^. The EOM can prepare the needed broadband signal, and there have been various proven techniques of chaotic signal generation and synchronization in electrical circuits. Indeed, recently experimental demonstration of chaos synchronization in a four-node optoelectronic network was reported^36^.

To show the efficiency of state transmission in q-CDMA, let us calculate the fidelities *F*_1_ = 〈*ϕ*_1_|*ρ*_3_|*ϕ*_1_〉 and *F*_2_ = 〈*ϕ*_2_|*ρ*_3_|*ϕ*_2_〉, where *ρ*_3_ and *ρ*_4_ are the quantum states received by atoms 3 and 4, and 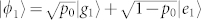 and 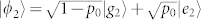 are the two quantum states to be transmitted. By designing the control parameters *g_i_* (*t*), using the Raman transition technique^18^, we find for the particular chosen quantum states that the fidelities *F*_1_ and *F*_2_ can be approximated as *F*_1_ = *F*_2_ ≈ 1 − *M*. When the Duffing oscillator enters the chaotic regime, we have *M* ≈ 0, leading to fidelities *F*_1_, *F*_2_ ≈ 1, which means that the qubit states can be faithfully transmitted over the q-CDMA network.

**Figure 4:**
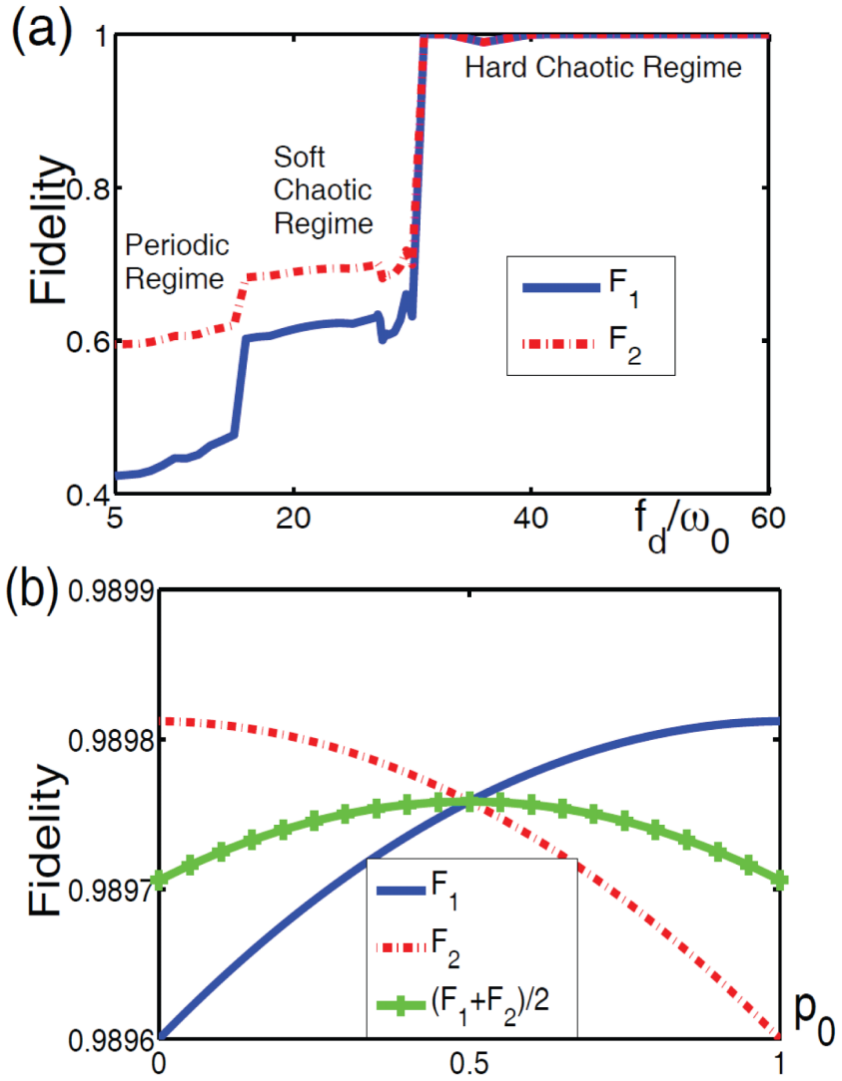
Fidelities of quantum state transmission. (a) Fidelities *F*_1_ and *F*_2_ versus the strength *f_d_* of the driving force acting on the Duffing oscillator with *p*_0_ = 0.6, and *τ* = 2*π*/*ω*_0_ as the unit of time. (b) *F*_1_, *F*_2_ and their average (*F*_1_ + *F*_2_)/2 versus *p*_0_ in the hard-chaotic region, with *f_d_*/*ω*_0_ = 36. The average fidelity is maximized at *p*_0_ = 0.5, which corresponds to |*ϕ*_1_〉 = |*ϕ*_2_〉.

We show the feasibility of the q-CDMA method using numerical simulations with the system parameters *ω_d_*/*ω*_0_ = 5, *g*_f–o_/*ω*_0_ = 0.03, *μ*/*ω*_0_ = 0.25, *γ*/*ω*_0_ = 0.05, *k_I_*/*ω*_0_ = 0.1, and *p*_0_ = 0.6. In Fig. 4(a), it is seen that there are three distinct regions representing how the chaotic motion affects the fidelity of the quantum state transmission. In the periodic regime characterized by 0 < *f_d_*/*ω*_0_ < 15, both *F*_1_ and *F*_2_ experience slight increases with increasing *f_d_*/*ω*_0_, with 0.4 < *F*_1_ < 0.5 and 0.6 ≤ *F*_2_ ≤ 0.64. At *f_d_*/*ω*_0_ = 15, the Duffing oscillator enters the soft chaotic regime which is indicated by a positive Lyapunov exponential *λ* ≈ 0.038 and a sudden jump in fidelities. In this regime, delineated by 15 ≤ *f_d_*/*ω*_0_ ≤ 33, both *F*_1_ and *F*_2_ are still below 0.7. The dynamics of the Duffing oscillator enters the hard-chaos regime at *f_d_*/*ω*_0_ ≈ 33, where both *F*_1_ and *F*_2_ suddenly jump to 1, which corresponds to an almost 100% faithful state transmission. In Fig. 4(b), we plot the trajectories of *F*_1_ and *F*_2_ as a function of *p*_0_ in the hard-chaotic regime *f_d_*/*ω*_0_ = 36, corresponding to *M* ≈ 0.0103. It is seen that *F*_1_ and *F*_2_ are very close to 1 − *M* ≈ 0.9897 and almost constant regardless of the value of *p*_0_. There are small deviations from 1 − *M*, because here *M*^2^ terms are not neglected. The average fidelity 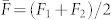 is maximum at *p*_0_ = 1/2, which corresponds to an equally-weighted superposition of the quantum states |*ϕ*_1_〉 and |*ϕ*_2_〉. In such a case, the crosstalk between the channels becomes minimum, inducing only a very slight disturbance on these indistinguishable states.

### Information transmission rates

Next we consider the maximum transmission rates of classical and quantum information over the proposed q-CDMA network, and compare them, under certain energy constraints, with the achievable bounds of transmission rates in a q-FDMA network and in quantum networks without any multiple access method (i.e., single user-pair network). Here the classical information transmission rates are calculated in terms of the Holevo information^37^,^38^ and the quantum information transmission rates are defined by the coherent information^39^,^40^,^41^. We assume that the frequencies allocated to different user pairs in the FDMA network are equally spaced such that the number of users is maximized and cross-talks between adjacent channels are suppressed. Moreover, we restrict our discussion to *Gaussian channels* and *Bosonic channels*, respectively for the transmissions of quantum and classical information.

**Figure 5:**
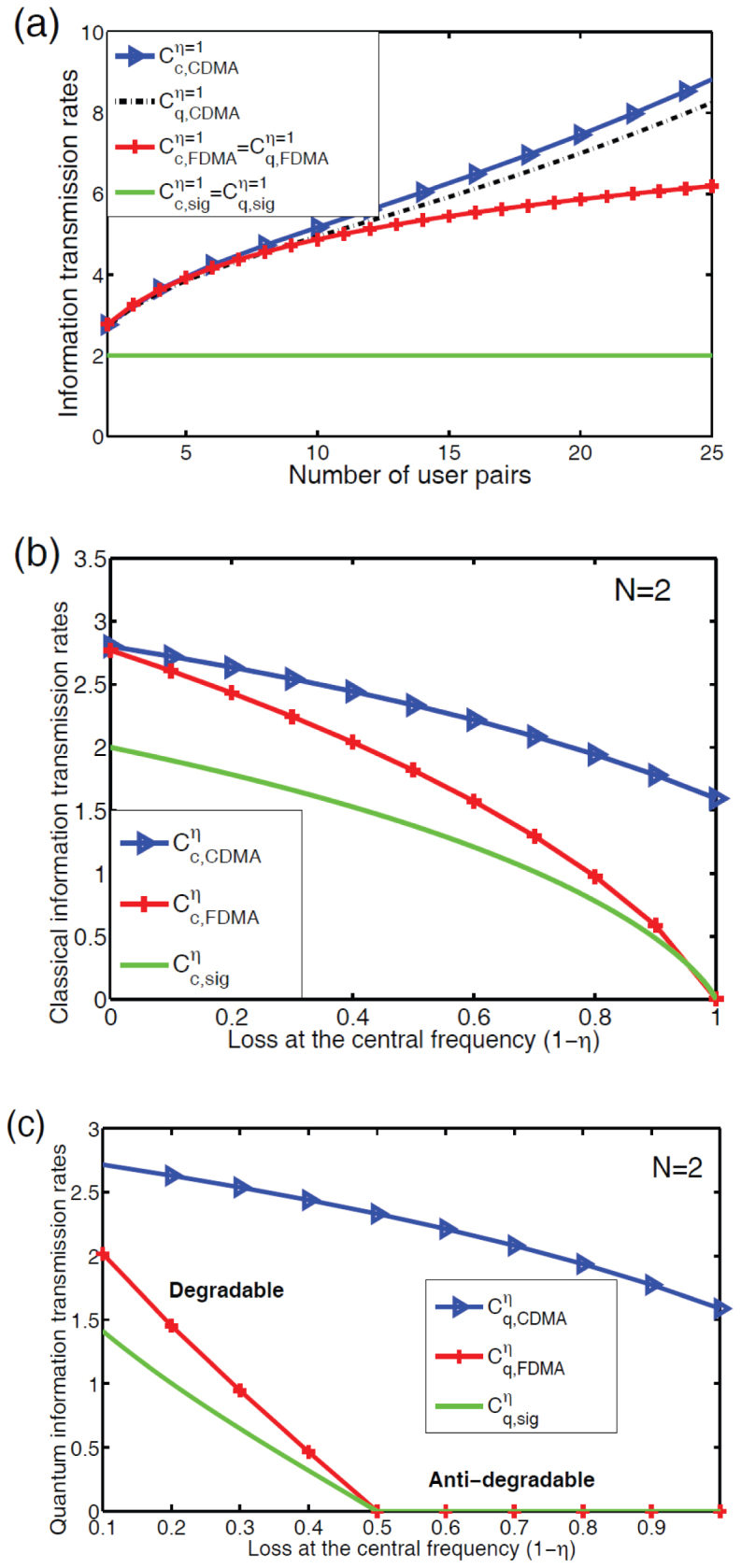
Quantum information transmission rates. (a) Fidelities *F*_1_ and *F*_2_ versus the strength *f_d_* of the driving force acting on the Duffing oscillator with *p*_0_ = 0.6, and *τ* = 2*π*/*ω*_0_ as the unit of time. (b) Upper bounds of the classical and quantum information transmission rates of different methods for ideal channel with *η* = 1 versus the number of the user pairs *N*. (c) and (d) Upper bounds of classical (quantum) information transmission rates of different methods for noisy channel with 0 < *η* < 1. The correction factor in the q-CDMA network is *M* = 0.01. FDMA is constrained by the frequency bandwidth *δω*/*ω* = 0.2. All the methods are constrained with the total energy 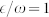. 
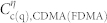
 denote the classical (c) and quantum (q) information transmission rates in q-CDMA and q-FDMA networks with transmissivity *η*. The rates for the single user-pair channel are 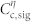 and 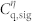.

We briefly summarize the main results here and in Fig. 5(a)–(c). (i) For lossless channels (i.e., *η* = 1 where *η* denotes the transmissivity of the central frequency of the information-bearing field), upper bounds of classical and the quantum information transmission rates for the proposed q-CDMA network are higher than those of the quantum FDMA and the single user-pair networks if the crosstalk in the q-CDMA is suppressed by setting 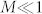. (ii) With the increasing number *N* of user-pairs in the networks, q-CDMA increasingly performs better than the q-FDMA for classical and quantum information. (iii) Information transmission rates for the q-CDMA is more robust to noise. For fixed *N*, quantum information transmission rates of the q-FDMA and the single user-pair networks degrades very fast to zero as the loss 1 − *η* increases from zero (ideal channel) to 1/2, whereas the q-CDMA network retains its non-zero rate even for very noisy channels. For the classical information transmission, the situation is similar except that the transmission rates of q-FDMA and the single user-pair network drops to zero when *η* = 0 which corresponds to a completely lossy channel.

The robustness of the proposed q-CDMA network for noisy channel can be explained as follows. The chaotic phase shifters in the q-CDMA network spread the information-bearing field across a broad spectral band. Thus, the energy distributed in a particular mode is almost negligible, and thus the photon loss is also almost negligible. Therefore, increasing *η* has very small effect on the transmission rates. In Fig. 5(b)–(c), we consider the noise to be broadband, and shows that the transmission rates of classical and quantum information over the q-CDMA network change only slightly.

## Discussion

We have introduced a q-CDMA network based on chaotic synchronization where quantum information can be faithfully transmitted with fidelities as high as 0.99 between multiple pairs of nodes sharing a single quantum channel. The proposed quantum multiple-access network is robust against channel noises, and attains higher transmission rates for both classical and quantum information when compared to other approaches. A q-CDMA network based on our proposal requires the realization of two important issues. First, quantum interference of signals from different chaotic sources. This has recently been demonstrated by Nevet *et. al*^42^. Second is the implementation of chaotic phase shifters and their synchronization. These could be implemented in various systems, including but not limited to optomechanical, optoelectrical^35^, and all-optical systems^33^. In particular, whispering-gallery-mode (WGM) optical resonators are possible platforms as chaotic behavior in a WGM microtoroid resonator has been reported in Ref^43^. Although synchronization of self-sustaining oscillations in directly coupled microring resonators have been demonstrated^44^, and mechanical mode synchronization in two distant resonators coupled via waveguides has been proposed^45^, demonstration of chaos synchronization in such optomechanical resonators are yet to be demonstrated. Although the tasks to be fulfilled are not trivial, we believe that we are not far away from such realizations due to the rapid pace of experimental and theoretical developments we have seen in the field in the past few years. We think that our proposal will pave the way for long distance q-CDMA networks, and will give new perspectives for the optimization of quantum networks.

## Methods

### Averaging over the chaotic phase shift

A chaotic signal *δ_i_*(*t*) can be expressed as a combination of many high-frequency components, i.e., 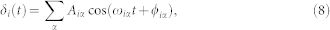
where *A_iα_*, *ω_iα_*, *ϕ_iα_* are the amplitude, frequency, and phase of each component, respectively. Then the phase of the signal at any given time *t* can be written as 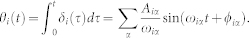


Using the Fourier-Bessel series identity^31^: 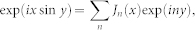


with *J_n_*(*x*) as the *n*-th Bessel function of the first kind, we can write 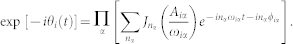


If we take average over the “random” phase *θ_i_*(*t*), the components related to the frequencies *ω_iα_* should appear as fast-oscillating terms and thus can be averaged out. This treatment corresponds to averaging out the components that are far off-resonance with the information-bearing field, and keeping only the near-resonance components. Hence, only the lowest-frequency terms, with *n_α_* = 0, dominate the dynamical evolution. Thus, we have 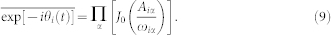
Since the chaotic signal *δ_i_*(*t*) is mainly distributed in the high-frequency regime, we have 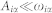. Using the expressions *J*_0_(*x*) ≈ 1 − *x*^2^/4, log(1 + *x*) ≈ *x* for 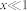, it is easy to show that 
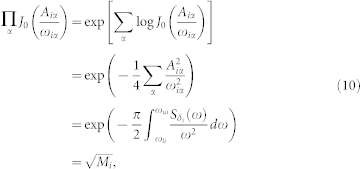
where 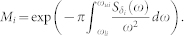


Consequently, from Eqs. (9) and (10), we obtain the equation 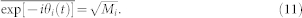


### Input-output relationship of the quantum CDMA network

**Figure 6:**
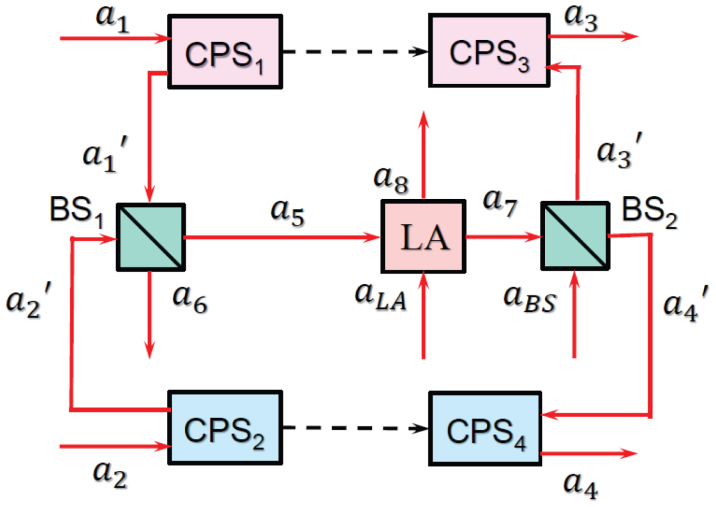
Input-output structure of quantum CDMA network. The black dashed lines denote the desired chaotic synchronization channel. The red lines show the quantum optical channels. “LA” refers to linear amplifier. “BS” refers to beamsplitter. “CPS” denotes chaotic phase shifter.

Here we calculate the input-output relationship of the quantum CDMA network shown in Fig. 6, we can express the input-output relationships of the chaotic phase shifters CPS*_i_*_ = 1,2,3,4_ as 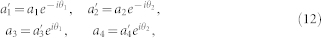
and those of the two beam splitters BS_1_ and BS_2_ and the linear quantum amplifier “LA”, respectively, as 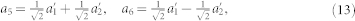

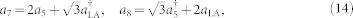
and 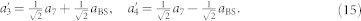
Then, using Eqs. (12–15), we obtain the total input-output relationship of the quantum network as 
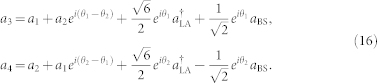
where *θ*_1_ and *θ*_2_ are independent chaotic “noises” as we have not considered chaos synchronization yet.

## Author Contributions

J.Z. proposed the main idea. J.Z., S.K.O., Y.X.L. and F.N. wrote the main manuscript text. R.B.W., F.F.G., X.B.W. and L.Y. participated in discussing the results and contributed to the findings of this paper.

## Figures and Tables

**Figure 1:**
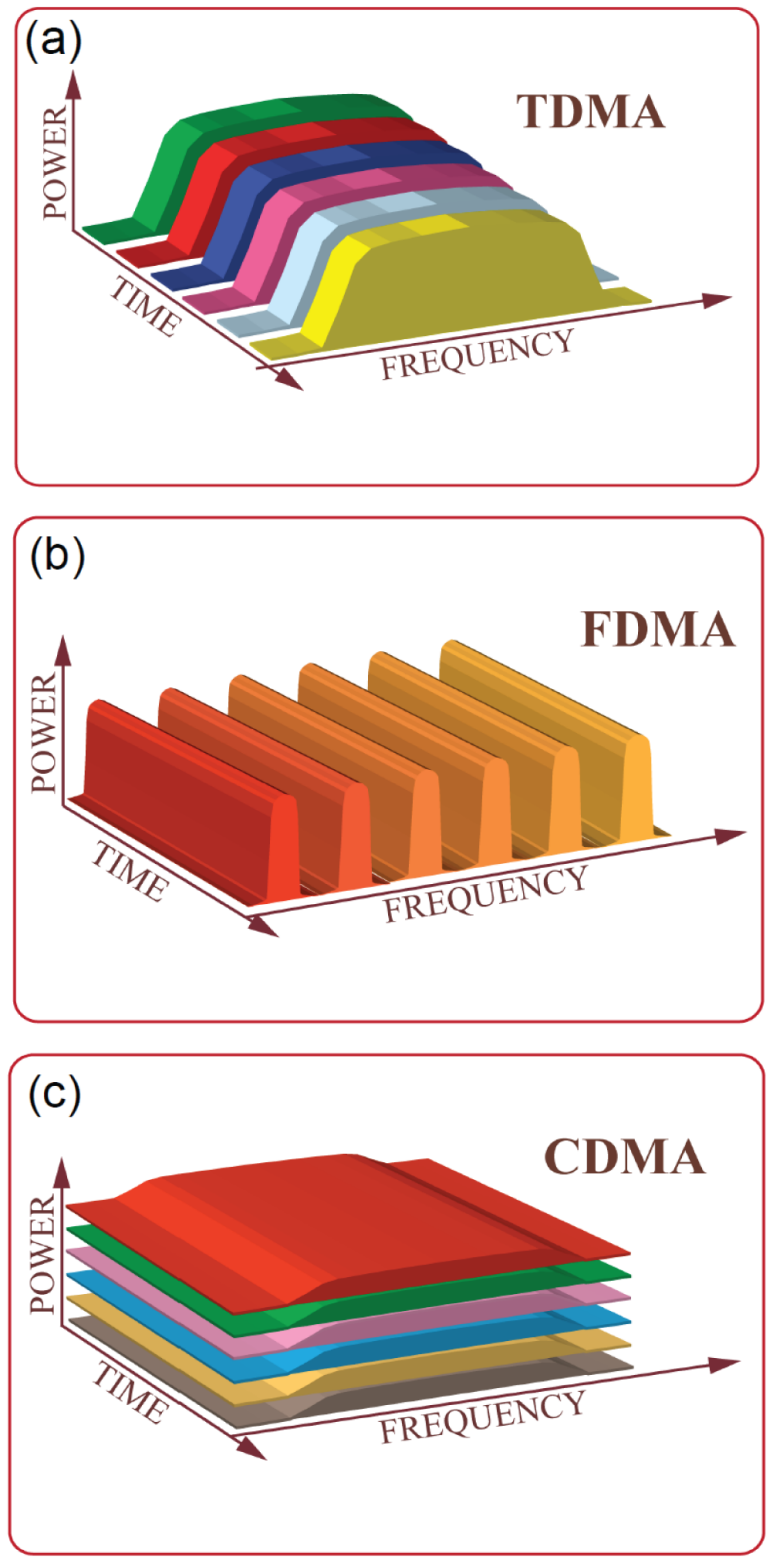
Illustration for different multiple-access methods. (a) TDMA: the users share the same frequency at different time slots. (b) FDMA: different frequency bands are assigned to different data-streams. (c) CDMA: the entire spectrum is utilized to encode the information from all users, and different users are distinguished with their own unique codes. Each user in the network is represented by a different color.
